# Psychosocial Outcomes of Supported Living for People with Severe Mental Illness: A One Year Evaluation of Floating Outreach in Germany

**DOI:** 10.1007/s10597-024-01400-5

**Published:** 2024-12-04

**Authors:** Lorenz B. Dehn, Julia Schreiter, Ingmar Steinhart, Martin Driessen

**Affiliations:** 1 Universitätsklinik für Psychiatrie und Psychotherapie, Evang. Klinikum Bethel, Remterweg 69-71, D-33617 Bielefeld, Germany; 2 Medical School OWL, AG 106 Psychiatrie, Bielefeld University, Psychotherapie, Psychosomatik, Bielefeld, Germany; 3 Institut für Sozialpsychiatrie Mecklenburg-Vorpommern, Universität Greifswald, Rostock, Germany

**Keywords:** Supported accommodation, Independent supported housing, Psychiatric rehabilitation, Social psychiatry, Needs

## Abstract

Supported living plays an important role in the community-based care for people with mental illness. However, support services like floating outreach have hardly been the subject of longer-term research to date, especially with regard to Germany. Thus, the main aim of this prospective observational study was to evaluate the psychosocial outcomes of floating outreach support for non-homeless people with severe mental illness across a one-year period. In a group of *n* = 119 people (M = 41 years old, 36% with affective disorders) the quantitative analyses revealed significant improvements in quality of life (MANSA), overall social functioning (SFS), as well as general support and care needs (CAN-EU). Nevertheless, there were still some unmet (and even increasing) care needs among the respondents after one year, especially in the domains of “physical health” and “company of others“. These findings therefore indicate areas of support that should be given more attention in the future.

## Introduction

Supported living is an essential element within the care and rehabilitation system for people with (severe) mental health disorders. Since the psychiatric deinstitutionalization processes of the last century, a wide variation of such housing-based support models have been developed (Farkas & Coe 2019, McPherson et al., 2018a). Although terminology and definitions for mental health supported living may vary transnationally, a number of common types have been established, especially in the western world, based on a traditional stepwise or gradual care model (see McPherson et al. 2018, Martinelli et al., 2019, Barbato et al., 2020, Harrison et al., 2020; Ketola et al., 2022): From *Residential care* providing intensive and long-term support with more or less 24 h staff presence, over *Supported housing* with staff on-site and time-limited support to independent supported housing or *Floating outreach*, which involves visiting support (a few hours per week) to service users living in their own, permanent tenancy. These characteristic types are also core components of the widely used simple taxonomy for supported accommodation (STAX-SA, McPherson et al. 2018). However, concepts and operationalizations may be somewhat different in other countries around the world, e.g. in low- and middle-income countries, where a significant need for research has also been described (Mhalange et al. 2024).

According to article 19 of the UN Convention on the Rights of Persons with Disabilities (United Nations, 2006), service users with (mental health) disabilities should have the possibility to choose the type of living form that best matches their individual needs. If persons with mental illness were asked, the majority favors an independent housing type, first of all, one’s own apartment (Piat et al., 2008; Richter & Hoffmann, 2017). In the meta-analysis by Richter and Hoffmann (2017), including data from *n* = 3134 service users, 84% of study participants with serious mental illness expressed a preference to live independently in their own home, with their family, or with people of their choice, whereas about one in five persons preferred to live in a more supervised accommodation setting. Moreover, there is also meta-analytic evidence that satisfaction with living conditions is better for people living in floating outreach compared to other supported accommodation types (Harrison et al., 2020).

According to official social welfare data from Germany (BAGüS 2022), the number of persons with (any form of) disabilities receiving floating outreach increased by about 69% between 2011 and 2020 (from *N* = 135436 to *N* = 228291), while the proportion of Residential Care users increased just by about 1.8% during the same period (from *N* = 190582 to *N* = 194010). This is partly due to the increasing de-institutionalisation of support services: In contrast to large residential, often remote facilities, community-based services are much more attractive and acceptable to many users (Wienberg, 2024). At present, people with (severe) mental or drug-related illnesses (compared to physical or intellectual disabilities) make up the largest user group of floating outreach support (70%) in Germany, as opposed to Residential Care setting (30%) (BAGüS 2022). The fact that the use of care services for mental illness has now become less stigmatising and that there is an growing number of providers of such services might play a role in this current utilisation rate (Wienberg, 2024).

To sum up, supported living in one’s own accommodation is particularly preferred by users, and is becoming increasingly widespread. However, while there is evidence that supported living is effective across a range of psychosocial outcomes for homeless and deinstitutionalized populations of people with severe mental illness, the need for more research on non-homeless psychiatric populations has been repeatedly pointed out (Chilvers et al., 2006, McPherson et al. 2018, Barbato et al., 2020, Killaspy & Priebe, 2021, van Genk et al., 2023). For instance, in their systematic review, Richter and Hoffmann (2017) included 32 studies that examined the outcomes of living in independent settings versus institutionalized accommodation for people with severe mental disorders. However, only eight of these studies covered non-homeless people, and out of these, only two were not from the USA or Canada. The only study from Germany at the time (Kallert et al., 2007), was based on people with (chronic) schizophrenia who lived in different types of supported housing. In fact, the recent study situation on housing interventions in Germany is relatively limited (Gühne et al., 2017, 2019), in particular with regard to nonresidential forms such as floating outreach (Walther, 2014). This research lack has been partly attributed to the fact, that the need for supported living due to (mental) health impairments must be granted according to the German social law requirements. However, whether these services were successful and purposeful at all seems to have played only a secondary role in the past (Walther, 2014).

On this background, the main aim of the present study was to evaluate the psychosocial outcomes of floating outreach support for non-homeless people with severe mental illness across a one-year period. The overall rationale of the study was based on the assumption that clients would experience positive changes in various areas of life during one year of floating outreach support. Although a wide variety of different outcome measures have been used in studies on supported living, quality of life, symptom severity, and social functioning are among the “most common” (McPherson et al. 2018). Additionally, in terms of mental-health outcome quality, researchers also argue that it is important to ensure that the services provided are tailored to the actual support needs of the clients (Gelkopf et al., 2022).

## Methods

### Study Setting

For the study’s purpose, the data from two similarly designed research projects were analysed in a combined secondary data analysis. These two study projects were carried out in different federal states of Germany, Mecklenburg-Western Pomerania1 and North Rhine−Westphalia2, and both aimed to investigate the outcome quality of the existing local floating outreach program. According to the Simple Taxonomy for Supported Accommodation (STAX−SA, McPherson et al. 2018), the floating outreach support addressed in the present study corresponds to the STAX−SA type 4, where service users are visited by support staff in their individual, permanent tenancy, and receive low to moderate support (a few hours per week). In Germany, people with mental health problems have a right for social support, including supported living. In most cases, the final decision on what type of support is needed and funded is made by the state social welfare agencies on the basis of a structured assessment and an evaluation of the person’s wishes. Each study project was funded by public, non−profit grants, organised in cooperation with local housing support providers and carried out by an external research institution. Despite different local conditions, the research institutions had previously co−ordinated a parallel study procedure and a comparable evalation methodology. The study procedures were approved by the local Institutional Review Boards (University of Muenster Ethics Committee: 2017−149−f−S, University Medicine Greifswald Ethics Committee: BB081/17).

### Participants

For inclusion in the present study, the following criteria had to be met: (A) age 18 to 69 years, (B) a severe mental illness, diagnosed by a psychiatrist, which lasted at least 2 years and (C) was associated with a functional impairment that constitutes the right for supported living according to the German social law IX, (D) new admission (or switch from Residential Care) to Floating Outreach support, (E) informed written consent for participation in the study, and (F) available assessment data from study entry (T1) and 12-month assessment (T2). Exclusion criteria were: (A) non-sufficient German language capacities, and (B) comorbid severe medical conditions (to avoid a confounding of functional and participation impairments caused by both somatic and psychiatric factors).

### Measures

Based on the study objectives, the following client-reported outcome measures were used as part of the assessment procedure at study entry and 12-month later:

*Social functioning* was assessed using the German version of the Social Functioning Scale (SFS) (Iffland et al., 2015). The original SFS was has been developed to assess social adjustment in schizophrenic patients (Birchwood et al., 1990), but its psychometric characteristics have been repeatedly confirmed in different psychiatric samples (Hellvin et al., 2010; Chan et al., 2019). Cronbach’s alpha of. 81 indicates a good reliability of the German versions full scale score. The 76-items self-assessment questionnaire covers a variety of aspects of social functions by 7 subscales (see Table 1), with higher scores indicating more competent social behavior or higher frequency (Iffland et al., 2015). The raw mean values of the subscales and the total sum score are reported here.

**Table 1:** Comparison of the outcome measures at study start (T0) and after 1 year (T1)

	T0 (study baseline):		T1 (after 12months):		Statistics ^#^	Effect size *
	M (SD)	Md	M (SD)	Md		
SCL-K9 (GSI mean score)	1.9 (0.9)	1.9	1.7 (1.0)	1.7	T(118) = 1.9, *p* =.063	d = 0.17
MANSA (total mean score)	3.0 (1.3)	3.0	3.3 (1.2)	3.4	T(118)=-4.4, *p* <.001***	d = 0.41
SFS (total sum score)	110.4 (21.2)	112	114.4 (21.6)	114	T(118)=-2.9, *p* =.005 **	d = 0.26
- SFS 1 withdrawal	9.1 (2.8)	9.0	9.5 (2.5)	9.0	Z=-1.7, *p* =.094	d = 0.33
- SFS 2 interaction	6.1 (2.1)	6.0	6.7 (1.9)	7.0	Z=-2.9, *p* =.003 **	d = 0.55
- SFS 3 independ. performance	28.6 (5.8)	29.0	29.5 (5.6)	31.0	Z=-2.3, *p* =.021 *	d = 0.43
- SFS 4 independ. competence	33.7 (3.5)	34.0	34.2 (3.9)	35.0	Z=-1.5, *p* =.123	d = 0.28
- SFS 5 recreation	18.3 (5.8)	18.0	18.7 (6.2)	18.0	Z=-1.2, *p* =.228	d = 0.22
- SFS 6 prosocial activities	10.9 (8.0)	9.0	11.4 (8.3)	10.0	Z=-1.1, *p* =.291	d = 0.20
- SFS 7 occupation	3.8 (3.2)	3.0	4.5 (3.1)	5.0	Z=-3.1, *p* =.002 **	d = 0.59
CAN-EU (total sum score)^+^	12.3 (5.6)	12.0	11.1 (5.2)	11.0	Z=-2.5, *p* =.012 *	d = 0.47
CAN-EU (no. of unmet needs) ^+^	3.7 (2.9)	3.0	3.4 (2.8)	3.0	Z=-1.6, *p* =.107	d = 0.30

^#^ paired T-Test or Wilcoxon-Test depending on normal distributed data according to Kolmogorov-Smirnov Test ^+^*n* = 110, * Cohens d

*Symptom severity* or subjective burden of psychopathological symptoms was assessed by the Symptom Checklist short form with 9 items (SCL-K-9) (Klaghofer & Brähler, 2001). This short form was developed from the original 90 items long version (SCL-90-R) by selecting the item from each of the original 9 subscales that had the highest correlation with the overall Global Severity Index (GSI-90). The resulting 9-item scale correlated with the GSI-90 at *r* =.93 and showed a Cronbachs alpha  =0.87 (Klaghofer & Brähler, 2001). The psychometric properties of the SCL-K9 were also verified in samples with psychiatric patients (Prinz et al., 2013) as well as in the general population (Petrowski et al., 2019). Here we report the Global Severity Index (GSI) as the total mean value of the 9 items.

*Quality of life* was addressed by the Manchester Short Assessment of Quality of Life (MANSA) (Priebe et al., 1999). Although this questionnaire was created primarily to assess subjective quality of life in patients with schizophrenia-spectrum disorders, a strength of this instrument is that the latent concept of quality of life measured is not specific to health-related issues (Petkari et al. 2020). The questionnaire covers the subject’s general satisfaction as well as that regarding 16 life domains (e.g. finances, friendships, leisure time, etc.) and is reported to have sufficient psychometric properties in people with mental illness (Björkmann & Svensson 2005). For instance, in a recent study including three samples of people with severe mental illness (total *n* = 806), Cronbachs alpha ranged from 0.78 to 0.85 (van Nieuwenhuizen et al., 2024).

*Care and support needs* were evaluated by means of the German-language version of the “Camberwell Assessment of Need - Europe” (CAN-EU, Kilian et al., 2001, McCrone et al., 2001). This interview-based measure takes into account 22 domains of individual care needs, including household, social contacts, physical health, daily activities, mobility, or social services etc. For each domain, the respondent is asked whether he/she had a need for support in the last month and to what extent this has been met. The domain scoring ranges from 0 (“no need”, because no problem in this domain) to 1 (“met need” or no/moderate problem due to help given) to 2 (“unmet need” or serious problem because no help is available). Research findings from over 2625 interviews of 1017 persons have shown that treatment outcomes in psychiatric practice can be quantified using the rate of change from unmet to met needs according to the CAN-EU results (Drukker et al. 2008). We report the total score as the sum of all domain scorings and the number of domains with unmet need.

### Statistics

According to the inclusion criteria it was possible to include *n* = 17 people from Mecklenburg-Western Pomerania and *n* = 102 people from North Rhine-Westphalia for the aim of the present secondary data analysis. However, in order to analyse these two samples together, their comparability had to be verified first. For this purpose, statistical group comparisons using Chi^2^-or paired t-Tests were carried out between the two samples with regard to various socio-demographic and clinical characteristics (see supplemental material). These analyses revealed no statistically significant differences (all *p* >.05, two-tailed), so that samples were subsequently analyzed as one overall group.

In the overall study sample (*n* = 119), the proportion of missing data on continuous outcome variables ranged from 0.8% (*n* = 1) to 5.0% (*n* = 6) across both assessment points. It could be assumed that this missing data was completely at random, according to Little’s missing completely at random (MCAR)-test (Chi^2^(156) = 169.24, *p* =.222). Thus, the missing values were imputed using the maximum likelihood-based expectation maximization (EM) procedure (Graham, 2009). Changes in the outcome measures between baseline (T0) and 12-month (T1) assessment were analyzed using paired t-tests or Wilcoxon-tests, depending on normal distributed data according to the Kolmogorov-Smirnov Test. In the case of significant results, associations of the change score (T1 minus T0) with basic variables (age, gender, work status, main diagnosis, prior supported housing experiences) were exploratively examined. To analyze the change in CAN-EU unmet needs (vs. met/no needs) over time, the McNemar test for dependent dichotomous variables was applied (Adedokun & Burgess, 2012). All statistical analyses were performed using the Statistical Software for the Social Sciences (SPSS Version 25). The general significance level was set to 0.05 and two-tailed.

## Results

### Sample Characteristics at Study Entry

An overview of the present samples demographic and clinical characteristics at study entry can be found in Table 2. The participants were on average 41 years old, two thirds have not been in employment, education or training at study entry, and while the average years in supported accommodation to date was around two years, half of the group had no prior experience with any form of supported accommodation (Md = 0 years). The most frequently occurring diagnosis was depressive disorders, and there were psychiatric and somatic comorbidities in two thirds of the participants.

**Table 2:** Baseline demographic and clinical data of the study sample (*n* = 119)

Study sample characteristics	Descriptive data
Age (in years)	M = 41.3 (SD 12.1) Md = 42, Min-Max: 20–69
Gender (female/male)	*n* = 64 (54%) / *n* = 55 (46%)
Completed vocational training (yes)	*n* = 63 (53%)
Currently not in education, employment or training	*n* = 78 (66%)
Years in supported accommodation to date	M = 2.4 (SD 4.6) Md = 0, Min-Max: 0–22
ICD-10 Diagnoses (according to medical documentation):	
F10-19 (substance use disorders)	*n* = 17 (14%)
F20-29 (schizophrenia/psychosis)	*n* = 14 (12%)
F30-39 (affective disorders)	*n* = 43 (36%)
F40-49 (anxiety, stress-, somatoform disorders)	*n* = 19 (16%)
F60-69 (personality disorders)	*n* = 15 (13%)
Other	*n* = 11 (9%)
Psychiatric comorbidity (yes)	*n* = 80 (67%)
Somatic comorbidity (yes)	*n* = 79 (66%)

### Changes in Psychosocial Outcome Measures

After receiving one year of floating outreach service in their own homes, the participants showed statistically significant improvements in subjective quality of life (MANSA, *p* <.001) and overall social functioning (SFS, *p* =.005), but only by trend with regard to self-rated psychopathological symptom severity (SCL-K9, *p* =.063). As can be seen in Table 1, the increase in overall social functioning was at subscale level associated with statistically significant improvements in the domains of “interaction” (*p* =.003), “independence performance” (*p* =.021), and “occupation” (*p* =.002).

Exploratory analyses showed that the improvements in quality of life (r_s_=-0.19, *p* =.042), overall social functioning (r_s_=-0.35, *p* <.001) and the subscale “independence-performance” (r_s_=-0.31, *p* <.001) were slightly, but significantly correlated with age. That is, the younger the participants were, the more likely they tended to show improvements in these outcome measures. Furthermore, participants without any form of education, employment or work at study entry showed a significantly greater improvement on the “occupation” subscale than people who have had some form of occupational activity (U = 970.5, Z=-3.3, *p* <.001). The “occupation” subscale refers to the current employment status or, in the case of people who are not employed, the subjective ability to work and job search efforts. Indeed, the proportion of people without any form of education, employment or work dropped from 66% at study entry (see Table 2) to 54% in the second assessment (data not shown). After all, the exploratory analyses did not reveal any further differences in the significantly changed outcome measures in relation to gender, occupational status, main diagnosis or prior supported housing experiences (all *p* >.05).

### Course of Support and Care Needs

The following results on the support and care needs (CAN-EU) are based on *n* = 110 persons with not more than of 4 missings across the 22 life domains, because *n* = 9 people had to be excluded due to an exceeded number of missing values across both time points (in 80 to 100% of the domains). As can be seen in Table 1, over the 12-months period there was a statistically significant reduction in the average total needs score from around 12 to 11 (*p* =.012). However, there was no significant change in the average number of domains with an unmet need for support (*p* =.107): At both time points, there were on average about 3 domains among all clients where serious problems were perceived (see Table 1). Here, the number of individual domains with unmet needs varied between 0 and 16 at the start of the study and between 0 and 12 after one year.

**Fig. 1:**
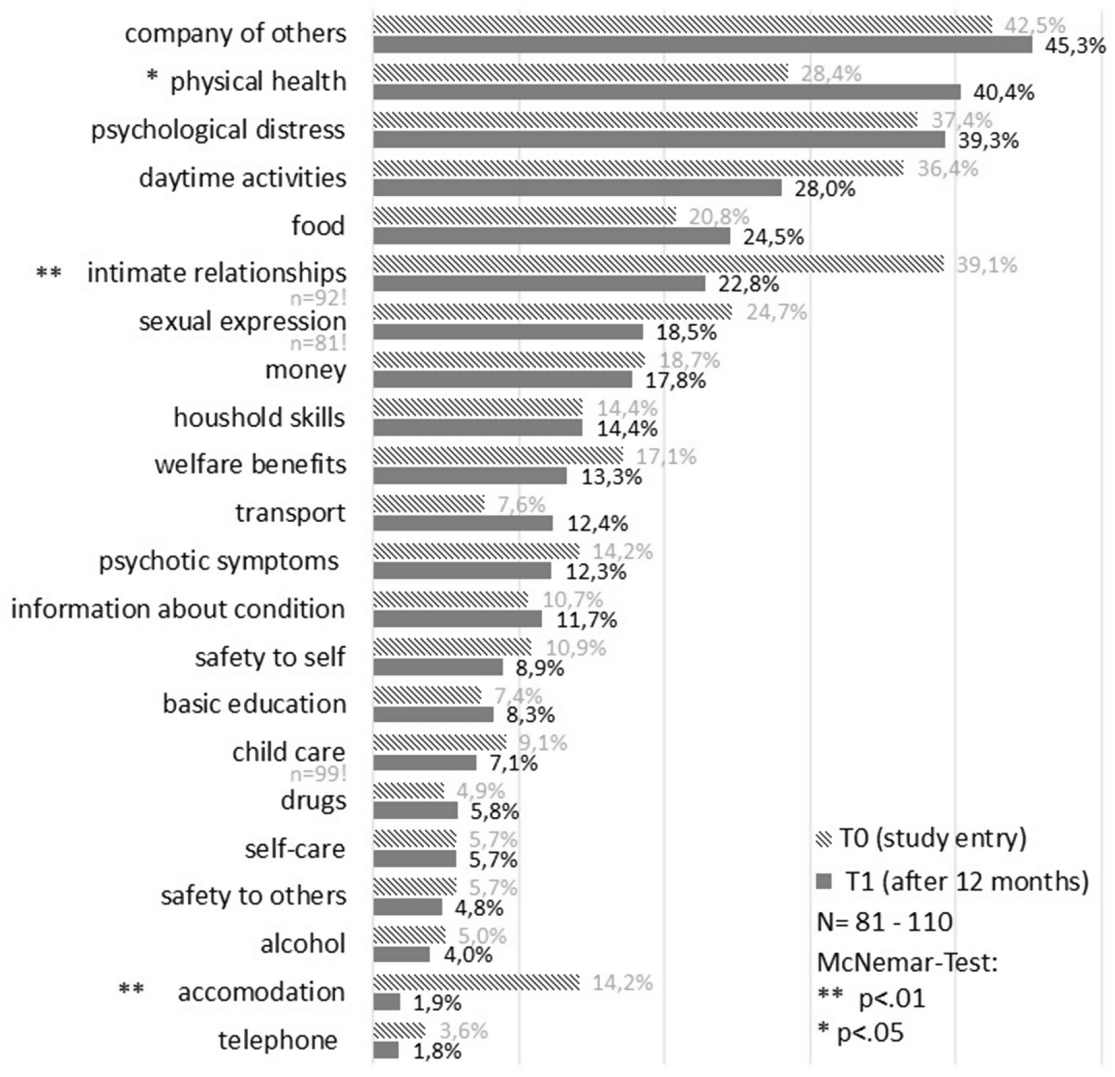
Proportion of unmet needs in the CAN-EU domains at study entry and 12 months later

A more detailed analyses of the domain-specific changes in unmet needs reveals the following results (see Fig. 1): In 12 of the 22 domains, the proportion of unmet need decreased between 1 and 16%, in two domains it remained exactly the same and it had increased between 1 and 12% in 8 domains. The strongest reductions of unmet needs in the two domains “intimate relationships” (-16%, *p* =.003) and “accommodation” (-10%, *p* =.007) were also statistically significant according to the McNemar test, as was the increase in the “physical health” domain (+ 12%, *p* =.035). Moreover, Fig. 1 shows that after a year, around one in two to three people experienced unmet needs in the domains of “company of others” (45%), “physical health” (40%) and “psychological distress” (39%). These three domains have already been among the most frequent problem areas at study entry (28 − 42%), but the (unmet) need for help in all three domains then even increased at the 12 months assessment.

In addition, exploratory analyses revealed, that the course of the support and care needs across the 12months study period had to be considered in relation to other client characteristics. For instance, there was a marginally significant relationship between the total needs change score and age (r_s_=0.212, *p* =.026), indicating that younger participants were more likely to show improvements. No differences were found here with regard to gender, occupational status, main diagnosis or prior supported housing experiences (all *p* >.05). However, gender was significantly related to the frequency of the unmet needs at the second assessment: Whereas such need for help in the area of “social contacts” was mentioned more often by women (55% vs. 34%, Chi^2^(1) = 5.0, *p* =.035), it was in turn more common among men in the “physical health” domain (51% vs. 31%, Chi^2^(1) = 4.9, *p* =.036). Unmet support needs regarding “physical health” at the 12months assessment were statistically not more frequent in participants with somatic comorbidity (44%) than in those without (32%, *p* =.228), although this group difference had in fact been statistically significant at the beginning of the study (36% vs. 13%, Chi^2^(1) = 6.5, *p* =.014).

## Discussion

The present study has shown, that in a sample of *n* = 119 clients with psychiatric illnesses who had newly started out housing-related support in their own homes, significant improvements in subjective quality of life, overall social functioning, as well as general support and care needs were seen in the follow-up assessment after 12 months. However, the perceived burden of psychiatric symptoms did not show any significant changes across all clients. In addition, after one year of housing support, clients continued to describe a high (and in some cases even increased) need for support in some areas of life, in particular company of others, physical health, and psychological distress. Against the background of insufficient empirical findings on the long-term outcomes of supported living for non-homeless people with psychiatric disorders, the present study results offer interesting insights into the psychosocial effects of a German floating outreach service.

### The Process of Recovery

The present results revealed different trajectories of the psychosocial outcome variables over the study period: While the psychopathological symptom severity has not been significantly reduced, and psychological distress necessitated a high level of help, the participants nevertheless showed a significant improvement in their general quality of life and in their psychosocial functioning and skills. With regard to the sub-areas of social functioning (Iffland et al., 2015), there were significant improvements in the client’s performance of skills necessary for independent living (e.g. using public transportation, household planning, going shopping without help), in their occupational situation (e.g. engagement in productive employment, a structured program of daily activity or the subjective ability to work and job search efforts), and in their self-perceived interaction behavior (number of friends/having a partner, quality of interpersonal communication). Thus, the present results show that the clients of floating outreach improve in coping with various areas of life and at the same time achieve an improved quality of life, despite persisting psychiatric symptoms and stress – which actually symbolizes the process of recovery. The concept of recovery does not focus on complete symptom resolution, but prioritizes the person themselves and emphasizes their resilience and control over problems and life (e.g. Jacob, 2015). Here, empirical research has confirmed that despite the presence of substantial psychiatric symptoms, a high degree of self-perceived recovery can be achieved and individual quality of life can be improved (Kukla et al., 2014). In this sense, our present results are in line with the conclusions of Krotofil’s (2008) systematic review on service user experiences of mental health supported accommodation, namely that “*As a home*,* supported accommodation can function as a base for recovery*…” (S. 796).

### Physical Health

Besides the different psychosocial improvements achieved, the present study also identified some unfulfilled needs of support among the floating outreach users, that need to be taken into account. Here, the domains “company of others”, “physical health” and “psychological distress” were not only among the most frequently mentioned unmet needs at study entry, but they were (still) experienced as a major problem by 39–45% of respondents in the 12-months assessment. Above all, the increase of unmet needs with regard to physical health is particularly striking, especially as there was not even (any longer) a significant association with somatic comorbidities after 12 months. This means that, regardless of accompanying somatic disorders, the issue of “physical health” represents a general care problem for which no support was reported in 40% of all clients. It is evident that individuals with mental illness are at an increased risk for a large number of physical health problems (De Hert et al., 2011). Moreover, the rates of undiagnosed and untreated medical illnesses are higher in (severe) mental illness, compared to the general population (Dornquast et al., 2017; Bradford et al., 2008). More recently, gaps in healthcare provision have also been described with regard to psychiatric rehabilitation, suggesting that more should be done here to address the persistent poor physical health of people with mental illness (Cockburn et al., 2022). Indeed, research has already shown that more intensive forms of supported living services can be an important mechanism for enabling people with psychiatric disorders to access the medical care they need (Nath et al., 2012). Thus, this seems to indicate that clients in floating outreach should be assessed and accompanied more explicitly with regard to physical health issues.

### Social Connectivity

In the present study, the area of life with the highest unmet need for help both at study entry (42,5%) and 12months later (45,3%) was “company of others”. This is also in line with the observed lack of significant changes in subjective social functioning with regard to “withdrawal” (social engagement, initiation of conversation etc.) and “prosocial activities” (e.g. engagement in common social activities like trips, cultural events or meetings with other people). Social isolation and feelings of loneliness are common among people with severe mental illness (Borge et al., 1999) and can be of major concern especially under more independent living arrangements (Fakhoury et al., 2002; Krotofil et al., 2018). For instance, in a cross-sectional study from Sweden among residents living in ordinary housing with support (Brolin et al., 2015), the factor “social life” yielded the lowest levels of satisfaction concerning housing situation and support. Of these residents, 21% reported that they were dissatisfied or very dissatisfied with their opportunities to socialize with other people (Brolin et al., 2015). In a systematic metasynthesis of experiences from people with mental illness living in support housing, Watson et al. (2019) found that these people typically reported feeling ‘cut off’, lonely and isolated, but also expressed a desire for relationships and a sense of belonging. Access to opportunities for social interaction and appropriate support were seen as helpful in reconnecting with family, friends and the social community (Watson et al. 2019). Social interaction has been found relevant for self-rated health and well-being not only in the general population (Mukerjee et al. 2013), but also among people with mental illness (Eklund et al. 2007). Taken together, our and other findings suggest that, much more attention should be given to tailor-made offers from healthcare professionals to support and initiate more social contacts in floating outreach clients.

### Limitations

When interpreting the present findings, some methodological limitations of this study have to be taken into account. First of all, the evaluation period only covers one year, while it should be considered that some psychosocial changes and recovery processes in persons with mental health illnesses may of course require a much longer duration. In the meta-analysis by McPherson et al. (2018), the follow-up periods across the *n* = 31 studies on homeless people with mental illnesses, where the strongest research evidence for mental health supported living comes from, ranged between 0,5 and 5 years, with an average of 1,8 years.

A further limitation concerns the characteristics of the present sample. While the socio-demographic data are overall comparable with other studies, e.g. with regard to age range or a high proportion of unemployed clients, there are nevertheless differences in the distribution of psychiatric diagnoses. Especially the proportion of people with schizophrenia/psychosis in the present study (12%) is much lower than in other related research projects e.g. de Heer-Wunderink et al. (2012), Killaspy et al. (2016) or Martinelli et al. (2019). Thus, the generalizability of the present results to other contexts might be limited.

Furthermore, it should be noted that the present analyses were carried out on the basis of pre-post comparisons of self-report measures. Such analyses are known to be susceptible to response shifts. This refers to a change in the meaning of self-assessments, as the meaning of constructs and outcome measures is time-dependent and alters after new life experiences (Vanier et al., 2021). A retrospective evaluation at the second assessment point would have opened up the possibility of obtaining a direct rating of change from the respondents’ perspective. Whilst these retrospective methods are also not free from bias (Blome & Augustin, 2015), this would allow better identification of what is important to participants and how this relates to the indirect measures of change.

## Conclusions

Over a period of one year, this study revealed improvements in the psychosocial situation of clients with mental health problems in connection with their floating outreach service. However, the present results also show that there are still unmet needs for help in various areas of life that should be given more attention. In addition to support in establishing social participation and fulfilling daily activities, the field of physical health in particular seems to require more professional attention, support and guidance. This could be grounded, for example, in a systematic and continuous assessment of the psychosocial conditions and needs of the people receiving support, in order to better align the supported living services to the individual requirements of the users.
